# Multi-Scale Coordination of Distinctive Movement Patterns During Embodied Interaction Between Adults With High-Functioning Autism and Neurotypicals

**DOI:** 10.3389/fpsyg.2018.02760

**Published:** 2019-01-11

**Authors:** Leonardo Zapata-Fonseca, Dobromir Dotov, Ruben Fossion, Tom Froese, Leonhard Schilbach, Kai Vogeley, Bert Timmermans

**Affiliations:** ^1^Plan of Combined Studies in Medicine (PECEM), Faculty of Medicine, National Autonomous University of Mexico, Mexico City, Mexico; ^2^Center for the Sciences of Complexity (C3), National Autonomous University of Mexico, Mexico City, Mexico; ^3^Research and High Performance Computing, LIVELab, McMaster University, Hamilton, ON, Canada; ^4^Department of Matter Structure, Nuclear Sciences Institute, National Autonomous University of Mexico, Mexico City, Mexico; ^5^Department of Computer Science, Institute of Applied Mathematics and Systems Research, National Autonomous University of Mexico, Mexico City, Mexico; ^6^Independent Max Planck Research Group for Social Neuroscience, Max Planck Institute of Psychiatry, Munich, Germany; ^7^Department of Psychiatry, Ludwig-Maximilians-Universität München, Munich, Germany; ^8^Department of Psychiatry and Psychotherapy, University Hospital Cologne, Cologne, Germany; ^9^Cognitive Neuroscience (INM-3), Institute of Neuroscience and Medicine, Research Center Jülich, Jülich, Germany; ^10^The School of Psychology, University of Aberdeen, Aberdeen, United Kingdom

**Keywords:** autism spectrum disorder, time-series analysis, social interaction, movement variability, human-computer interface, tactile interaction, social motor coordination, multi-scale analysis

## Abstract

Autism Spectrum Disorder (ASD) can be understood as a social interaction disorder. This requires researchers to take a “second-person” stance and to use experimental setups based on bidirectional interactions. The present work offers a quantitative description of movement patterns exhibited during computer-mediated real-time sensorimotor interaction in 10 dyads of adult participants, each consisting of one control individual (CTRL) and one individual with high-functioning autism (HFA). We applied time-series analyses to their movements and found two main results. First, multi-scale coordination between participants was present. Second, despite this dyadic alignment and our previous finding that individuals with HFA can be equally sensitive to the other’s presence, individuals’ movements differed in style: in contrast to CTRLs, HFA participants appeared less inclined to sustain mutual interaction and instead explored the virtual environment more generally. This finding is consistent with social motivation deficit accounts of ASD, as well as with hypersensitivity-motivated avoidance of overstimulation. Our research demonstrates the utility of time series analyses for the second-person stance and complements previous work focused on non-dynamical and performance-based variables.

## Introduction

Autism Spectrum Disorder (ASD) can be understood as a social interaction disorder (American Psychiatric Association and American Psychiatric Association DSM-5 Task Force, 2013). This makes the emerging “second-person approach” to social cognition a more promising framework for studying ASD than classical approaches focusing on mind-reading capacities in isolated, detached and observer-based arrangements (Gallagher, 2004; De Jaegher, 2013; Froese et al., 2013). According to this second-person approach, a variety of embodied, perceptual and interactive capabilities are required for the full competence of understanding others (Froese and Di Paolo, 2011; Schilbach et al., 2013; Froese, 2018), all of which are hypothesized to be compromised in ASD (Gallagher, 2004; Fuchs, 2015; Schilbach, 2016). This approach states that, rather than making sense of others through observation or simulation, which occur in the observer, we need to engage with the other person by means of reciprocal action control (Kunde et al., 2018), thereby not simply “understanding” the other, but rather to jointly engage in a set of learned social skills that give us an immediate attunement to the other.

We therefore investigated the capacity to detect social contingencies as the mutual responsiveness of interaction partners in 10 dyads of adult participants engaging in computer-mediated embodied social interaction. Each dyad consisted of one control individual (CTRL) and one individual with high-functioning autism (HFA). Participants were asked to distinguish the interaction partner from other non-reactive items by clicking. In previous work, we studied the accuracy of this social contingency detection, finding that individuals with HFA did not differ significantly in their clicking accuracy from controls (Zapata-Fonseca et al., 2018). However, individuals with HFA were notably more conservative in their clicking, and visual inspection of their movements revealed marked differences in style compared to controls. These observations in conjunction with recent work on stochastic patterns of motor variability to objectively characterize the ASD phenotype (Torres et al., 2017) motivated us to present this quantitative description of movement patterns during real-time embodied interactions. Our work aims to provide a quantifiable link between on the one hand individual motor movement markers as potential diagnostic tools for ASD (e.g., Cook et al., 2013), and on the other hand social interaction deficits of ASD, which are currently the two main diagnostic criteria.

## Materials and Methods

The time series of embodied interaction were recorded by means of a minimalistic human-computer interface paradigm that has become known as the “Perceptual Crossing Experiment” (PCE), originally developed by Lenay (2008); for a review, see Auvray and Rohde (2012). The real-time sensorimotor features of the PCE have proven to be capable of eliciting meaningful social interactions and forms of mutual alignment that are also found in real-life social interactions (Froese et al., 2014; Froese and Zapata-Fonseca, 2017).

### Participants

Participants within one dyad were matched with respect to sex and age. The 10 HFA participants (5 male) were between 29 and 54 years of age (*M* = 42.32, *SD* = 9.20) and were diagnosed and recruited at the Autism Outpatient Clinic at the Department of Psychiatry of the University Hospital of Cologne in Germany (one participant was diagnosed at the Cologne Autism Therapy Centre). The diagnoses were confirmed by clinical interviews according to ICD-10 criteria by two specialized physicians and were supplemented by extensive neuropsychological assessment. The sample included patients with the diagnosis “Asperger syndrome” according to ICD-10 with an at least average Full-Scale IQ (FSIQ N85, measured using Wechsler Adult Intelligence Scale, WAIS).

### Perceptual Crossing Experiment (PCE)

**FIGURE 1:**
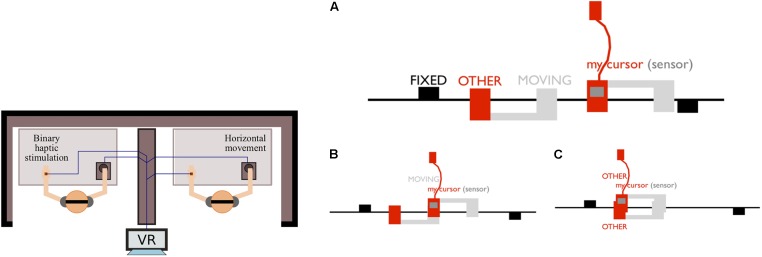
Experimental set up of the Perceptual Crossing Experiment. (Left) Two participants are physically separated and interact within a shared virtual space via a Human-Computer Interface consisting of a mouse and a tactile stimulator (illustration modified from Froese and Zapata-Fonseca, 2017). (Right) Illustration of a one-dimensional virtual space, taken from Zapata-Fonseca et al. (2018): **(A)** participants are embodied as avatars on an invisible line that wraps around after 600 pixels in a continuous fashion. Each avatar is controlled by a mouse and attached to a tactile feedback device; **(B)** in a situation of one-directional coupling, a participant interacts with the other’s shadow object, which moves identically to the other’s avatar, but without the other receiving feedback; **(C)** a mutual encounter is defined as the overlap between avatars, and therefore both participants simultaneously receive the tactile feedback.

During the PCE, two participants are mutually interacting as embodied avatars within a one-dimensional circular virtual space. Participants control the movement of their respective avatars using a computer mouse and receive information about a direct contact with objects in the virtual space via on-or-off tactile feedback to the hand. They can encounter three types of objects: (i) the other’s avatar; (ii) a moving object that “shadows” the other’s avatar; and, (iii) a static object (see Figure 1).

The task is to move around freely and to identify the partner’s avatar by clicking the mouse button to report a perceived encounter, but not during encounters with either the shadow or the fixed object. The three types of objects produce identical sensory consequences to the hand when a participant’s avatar meets them in the virtual space but provide different affordances (potentials) for interaction. In particular, distinguishing between a static object, the partner’s moving avatar, and the shadow of a partner’s avatar depends on being sensitive to their particular affordances for interaction; only the other’s avatar can be responsive. Each dyad performed three trials of 5 min each. Participants do not receive feedback about their clicking performance until after the experiment.

### Time-Series Analysis of Participants’ Movement Patterns

In the PCE, the computer-mediated participants’ behavior permits a detailed recording of how their movements and sensations change over time; see Supplementary Figure 2. Time-series approaches to embodied social cognition shift the research question toward *how* rather than *what* tasks involving social capacities are being solved (Zapata-Fonseca et al., 2016; Romero et al., 2018).

#### Interpersonal Coordination

Complexity Matching (CM) accounts for generalized coordination between two multi-scale processes. CM is the fit between the power-laws describing how the variance of each process increases as the scale over which it is measured increases. CM is maximized when information exchange between the processes is maximized (West et al., 2008). For point processes, such as the zero-crossings of avatars’ acceleration, the variance is obtained using the Allan Factor (Allan, 1966). This method has been applied to groups of healthy participants (Abney et al., 2014; Zapata-Fonseca et al., 2016). Testing for complexity matching consists of surrogate analysis; see Supplementary Material’s Section 1.

#### Individual Movement Profiles

To study participants’ amount of movement and its variability (Torres et al., 2013; Słowiński et al., 2016), we took the magnitude and standard deviation of the velocities. Velocity was defined as the rate of change in positions (Zapata-Fonseca et al., 2016). From the velocity time series we took the absolute values and analyzed the mean (Speed_Mean) and the standard deviation (Speed_SD), each of which was fitted independently against *Group* and *Trial* using hierarchical modeling (Singer and Willett, 2003) in *lme4* (Bates et al., 2015); see Supplementary Material’s Section 2.

#### Multi-Scale Movement Variability

A more detailed study of the movement variability was made through coarse-grained analysis similar to Fossion et al. (2017). The time series is analyzed at different scales without decomposing it into different non-overlapping components, but instead incrementally filtering out finer scales such that the coarse-grained time series at finer scales will still contain the coarse features and only at the coarser scales the finer details will be filtered out (see Supplementary Figure 3). This allows to quantify how much small-scale and especially large-scale components account for the variability of a signal by computing its variance according to different resolution factors. In the context of the PCE small-scale fluctuations correspond to jittery movements, whereas large-scale components indicate sustained movements in one direction; see Supplementary Material’s Section 3.

## Results

### Complexity Matching Between Controls and Patients With HFA

Complexity matching between participants was present as revealed by the surrogate dyads analysis: D_a,b_^Original^ (*M* = 12.84, *SD* = 3.3) was significantly higher than D_a,b_^Surrogate^ (*M* = 11.35, *SD* = 1.06), *t*(29) = 2.46, *p* = 0.01 (see Supplementary Figure 1). The difference between similarity indices (D_a,b_^Original^ vs D_a,b_^Surrogate^) means that the structure of the movements’ variability across time scales was globally coordinated between the real dyads (participants).

### Speed Profiles Distinguish Between Controls and Patients With HFA

**FIGURE 2:**
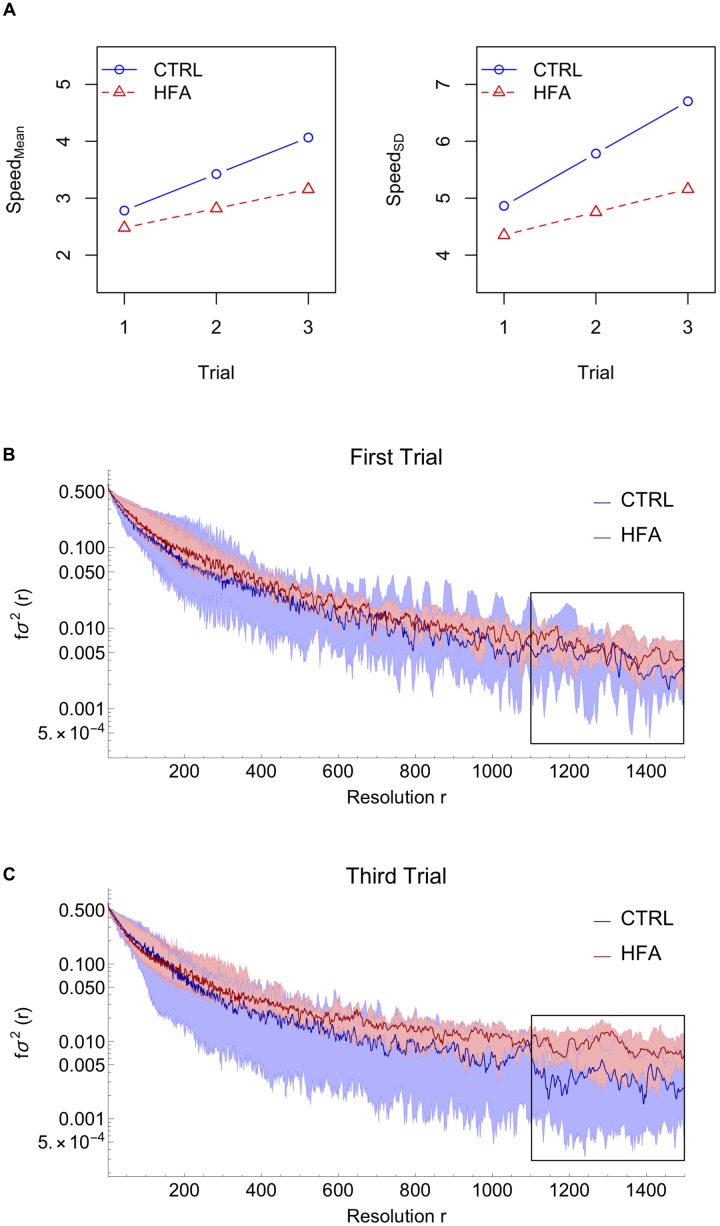
Results. **(A)** Model fit for the mean of speed (absolute velocity) and the standard deviation of speed. **(B,C)** Coarse-graining analysis results: Median (dark curves) and region between 1st and 3rd quartiles (shaded areas) for controls (CTRL) and high-functioning autism individuals (HFA). Comparing the end (trial 3) with the start (trial 1) of the experiment, we find that HFA participants start to deviate from CTRL participants, especially for r = 1100 to 1500, i.e., an approximate temporal resolution of 22 to 30 s.

For both dependent variables the significant coefficients can be found in Supplementary Table 1. Figure 2A shows the form of the fitted model: whereas numerically the CTRL group appears to have both higher Speed_Mean and Speed_SD than the HFA group, the factor *Group* did not contribute significantly to a model including *Trial* and the *Trial:Group* interaction as predictors, so, overall, the average Speed_Mean and Speed_SD did not differ between groups. Instead, there was a main effect of *Trial* (*p* = 0.059), for Speed_Mean, showing that mean speed increased over trials, without a significant interaction with *Group*. For Speed_SD on the other hand, there was no main effect of Trial, but there was an interaction between *Trial* and *Group* (*p* = 0.049), suggesting that only for CTRL did Speed_SD (variability in movements) increase over trials.

### Variability Pattern as Behavioral Marker in Patients With HFA

The coarse-grained analysis was sensitive to the participants’ different modes of movement understood as different variance values according to different time-scales. The fractional variance f*σ*^2^ decreased with increasing resolution factors *r*, meaning that all participants made more frequently smaller jittery movements than slow ones with large periodicity.

Figures 2B,C show that particularly in the third trial, the HFA group invests more variance in unidirectional and sustained movements (bigger *r-*values) when compared to the CTRL group. Notice that for the first trial the median values of both groups correspond, whereas for the third trial the median values deviate for coarser resolutions (*r* from 1100 to 1500, corresponding with an approximate temporal scale of 22 to 30 s). The statistical analysis performed for each *r* showed that from trial 1 to trial 3, HFA individuals progressively diverged from CTRL individuals (Supplementary Figure 4.1); further analysis of trial-to-trial changes per group showed to be due to HFA individuals progressively investing more in large-scale movements and less in small-scale jittery movements (Supplementary Figure 4.2).

## Discussion

In this report, we presented a quantitative characterization of the movement patterns during a social contingency detection task, systematically comparing the movement of HFA and non-HFA participants who could interact with each other. Such “movements perspective” (Torres et al., 2013) has been used to develop behavioral markers, particularly when the social component is compromised, like in ASD (Torres et al., 2016; Whyatt and Torres, 2017b; Ryu and Torres, 2018). Our findings further support this perspective of taking motor aspects as proxies of social deficits.

Our results showed that an overall mutual coordination was reached at the dyad level, even in this highly constrained, minimal environment. This so-called complexity matching between participants during real-time interaction is consistent with our previous findings in non-autistic participants: *D*_a,b_^Original^ = 12.47, and *D*_a,b_^Surrogate^ = 11.78 (Zapata-Fonseca et al., 2016). Also, it goes in line with findings of embodied social competence during conversation in children with ASD (Romero et al., 2018). Additionally, our research supports the usage of computer-mediated and tactile interactions for understanding the relationship between movement-based coordination and social engagement in patients with ASD (Dautenhahn, 2007; Robins and Dautenhahn, 2014). We suggest that technology might fulfill a scaffolding role (Aresti-Bartolome and Garcia-Zapirain, 2014; Pennisi et al., 2016) at the level of movement coordination, but future research is required to disentangle the specific factors leading to such multi-scalar coordination.

Despite the mutual coordination, the individual’s movement profiles showed differences between CTRL and AUT participants in relation to the trial number. Non-HFA participants increased their velocity of their movements much faster and in a more varied, if not “erratic” or unpredictable fashion across trials. The higher movement variability is consistent with the so-called *healthy range of variability*, not only in motor behaviors (Riley and Turvey, 2002; Romero et al., 2018), but also in other adaptive behaviors (Torres, 2013; Dotov et al., 2017; Dotov and Froese, 2018) and physiology dynamics (Rivera et al., 2016; Fossion et al., 2018). This suggests that non-HFA participants were less inhibited and more flexible in adapting their behavioral repertoire. Conversely, HFA participants consistently spent more time on large-scale movements and divested in small-scale jittery movements such as present during a mutual encounter, which indicates a preference for a more thorough, systematic and rational exploration of the virtual environment. Perhaps they focused on the social contingency detection task of clicking correctly, and hence less on engaging in social interaction for its own sake, whereas non-HFA persons appeared to “search for” and “enjoy” their partner, as has been anecdotally reported in other PCE studies (Auvray and Rohde, 2012). This interpretation is consistent with the reduced social motivation associated with autism, whereas the avoidance of jittery encounter movements and associated tactile stimulation may be related to ASD and hypersensitivity (Bottini, 2018; Clements et al., 2018).

## Conclusion

In conclusion, our previous analysis showed that the HFA group was sensitive to the presence of the other, as shown by their clicking accuracy when identifying the other (Zapata-Fonseca et al., 2018). The present analysis found that the implicit movement coordination was also comparable with the complexity matching found in non-HFA pairs (Zapata-Fonseca et al., 2016). However, our results also suggest that HFA participants distinctively avoided situations of mutual perceptual crossing and instead preferred an objective perceptual strategy. This is consistent with gaze studies showing that HFA individuals tend to actively avoid mutual gaze during interaction and instead prefer to perceptually explore the rest of the other’s body and environment (Guillon et al., 2014; Chevallier et al., 2015). Here, for the first time, we have revealed a similar pattern in the tactile modality.

Finally, our findings support previous research on movement profiles and their variability in ASD (Brincker and Torres, 2013; Torres and Denisova, 2016; Torres and Whyatt, 2017; Whyatt and Torres, 2017a, 2018), as well as on the value of the PCE and time-series analysis for quantitatively describing behaviors related to social cognition (Bedia et al., 2014; Zapata-Fonseca et al., 2016; Kojima et al., 2017). However, this work should be considered as preliminary because it is the first study to analyze the dynamic trajectories during this task in HFA, and motor and timing of movement deficits in ASD might be potential contributing factors to the effects found in this research and therefore require further studies. Importantly, the sample size was rather small and CTRL-CTRL pairs were not included here, so further analyses are required to clarify the contribution made by each individual to the interaction dynamics.

## Ethics Statement

This study was carried out in accordance with the recommendations of the guidelines of the Ethics Commission of the Medical Faculty of the University of Cologne (Ethik-Kommission der Medizinischen Fakultät der Universität zu Köln) with written informed consent from all subjects. All subjects gave written informed consent in accordance with the Declaration of Helsinki. As the HFA volunteer participants were former patients of the Poliklinik für Psychiatrie und Psychotherapie, University Hospital of Cologne, their personal data are stored on the password-protected intranet (without access to the internet) of the University Hospital of Cologne. For screening purposes, test data from participants were retrieved, but were anonymised. The protocol was approved by the Ethics Commission of the Medical Faculty of the University of Cologne.

## Author Contributions

BT, KV, and LS conceived the study. BT designed and performed the experiments. LZ-F, BT, RF, and DD analyzed the data in close discussion with TF. LZ-F, DD, and BT wrote the first draft of the manuscript. All authors have made a substantial, direct and intellectual contribution to the work, and approved it for publication.

## Conflict of Interest Statement

The authors declare that the research was conducted in the absence of any commercial or financial relationships that could be construed as a potential conflict of interest.
