# Supplementary material for: Multi-Scale Coordination of Distinctive Movement Patterns During Embodied Interaction Between Adults With High-Functioning Autism and Neurotypicals
Source: Front Psychol. 2019 Jan 11;9:2760. doi: 10.3389/fpsyg.2018.02760 (PMC6336705; doi:10.3389/fpsyg.2018.02760)
Supplement: Supplementary file 1 [file Data_Sheet_1.pdf]

Supplementary Material

Multi-scale Coordination of Distinctive Movement Patterns During Embodied Interaction Between Adults With High-Functioning autism and Neurotypicals

Leonardo Zapata-Fonseca *, Dobromir Dotov, Ruben Fossion, Tom Froese, Leonhard Schilbach, Kai Vogeley and Bert Timmermans

***Correspondence should be addressed to:**

- Leonardo Zapata-Fonseca. Plan of Combined Studies in Medicine (MD/PhD), Faculty of Medicine, National Autonomous University of Mexico, Mexico City, Mexico; zapatafonseca.leonardo@gmail.com

1. Complexity Matching

The time series from the Perceptual Crossing Experiment (PCE) are characterized by onsets and endings of activity. Therefore, the behavioral patterns can be characterized by a binary series of changes in movement direction, *i.e.* as sign changes in the velocity time series (zero-crossings). The statistical properties of the resulting point process are given by the distribution of inter-event intervals (IEI) and approximated the theoretical power-law exponent of two.
As in the previous study (Zapata-Fonseca et al., 2016), we took the data set of thirty bivariate trials (*N=30*, three sessions and ten dyads).

Complexity Matching (CM) was evaluated in terms of the overlap between the partners' scaling functions for point-process variance Allan Factor (AF) with respect to cluster size T. Allan factor is a form of variance for point processes. Consequently, complexity matching compares the power-law clustering in two point-processes by comparing their scaling functions.

AF variance $A(T)$ was obtained as follows. First, the signal is segmented into M adjacent windows of size T, determined by the number of non-overlapping windows covering the time series. Second, the number of events N_j is counted within each window M. The events are indexed by $j = 1$ to $j = M$. Third, a ratio similar to a coefficient of variation is obtained. The expected value of the squared differences $d(T) = N_{j+1}(T) - N_j(T)$ in counts between adjacent windows of a given size T is normalized by the mean counts of events per window. This is repeated across a range of cluster sizes T

$$A(T) = \frac{\langle d(T)^2 \rangle}{2\langle N(T) \rangle}$$

The power-law $A(T) \sim T^{\alpha}$ indicates a Poisson process when $\alpha = 0$, i.e. $A(T) \sim 1$ for all T, whereas if $\alpha >$ 0 it means that the distribution of the clustering follows a different power-law.

In the present study, the window size T ranged from .037 to 2.34 seconds. We also considered larger windows but the power-law linking AF to window size dropped down, suggesting an absence of variability on those scales, *i.e.*, no large clusters of zero-crossings beyond several seconds. To

determine complexity matching between two participants in a given session, a distribution similarity index is calculated by comparing the respective AF functions,

$$D_{a,b} = -\sum_{T} \log |\ A(T_a) - A(T_b)\ |$$

Testing for complexity matching consists of a surrogate analysis, in which surrogate dyads are constructed by scrambling the pairs, that is matching the signals from participants from different pairs, and then comparing their complexity matching scores $D_{a,b}^{\text{Surrogate}}$ with the original $D_{a,b}^{\text{Original}}$ values using a t-test (Zapata-Fonseca et al., 2016). ***Supplementary Figure 1*** shows the results for the aforementioned analysis.

2. Linear Mixed-Effects Modeling: Mean and Standard Deviation of Speeds

The sampling rate of the recordings was 20 milliseconds, yielding time series of 15,000 data points. (***Supplementary Figure 2***). The absolute value of the velocity (speed) was considered for this analysis focusing on the magnitude rather than on the sign of the time series.

For the dependent variables (Speed_Mean and Speed_SD) a minimal model consisting of a constant intercept*, i.e.,* a grand mean, was expanded by including the predictors trial number (*Trial*), clinical condition (*Group*), and their interaction targeting the same model template for consistency and easier interpretation. This technique was developed for the statistical analysis of longitudinal studies with multilevel designs (Singer and Willett, 2003). It resembles the regression of an outcome variable against multiple predictors and additionally can deal simultaneously with predictors at different levels, *i.e.,* time-varying predictors as well as constant randomly assigned grouping factors such as participant identity (pair number in the present study).

In the current study, six models were tested for both dependent variables; model #5 was the optimal model for both of them (see ***Supplementary Table 1***). The goodness of fit tests for each model are based on with Maximum-likelihood-estimated coefficients, which are comparable (but not equal) to regression slopes and intercepts estimated using least-squared-error.

3. Coarse graining

The variance of a time series (σ^2) is a single number which expresses the average square distance from all the data points to the average $\bar{x}$.

$$\sigma^2 = \frac{1}{N}\sum_{i=1}^{N} \left(x_i - \bar{x}\right)^2$$

One can look at the time series at different resolution scales r, by averaging the time series in successive non-overlapping windows of length r and replacing all the data points in a window by their average, in an operation which is called *coarse graining* (similar to (Fossion et al., 2017). At coarser resolution scales*, i.e.,* for larger r, variance tends to be less because small fluctuations are cancelled out and only the average behavior is left (see ***Supplementary Figure 3***). It is of interest to see how much each scale r contributes to the variance of the original time series, *i.e.,* we can calculate the fractional variance $f\sigma^2\ (r)$ of each scale with respect to the variance of the original time series,

$$f\sigma^2(r) = \sigma^2(r) / \sigma^2(1) \leq 1$$

For statistical analysis, we studied how the values for $f\sigma^2$ changed according to resolution factors *r*. So, for each resolution factor *r*, a Mann-Whitney test was applied for comparing either two samples of $f\sigma^2$ (*r*) values from different groups (CTRL and HFA) but for the same trial, or two samples of $f\sigma^2$ (*r*) values from the same group (CTRL or HFA) but for two different trials (see ***Supplementary Figure 4***). If the behaviors of the effect sizes are consistent over neighboring resolutions *r*, then it is considered as an indication that individual scales *r* are not independent but grouped together in *"components"*. We caution that there may be multiple comparisons issues; however, we also note that whereas a significant difference between groups on a single resolution factor can be attributed to a Type I error, observing such a difference for adjacent resolution factors is unlikely due to such an error, in that true Type I errors should be randomly distributed across resolution factors.

4. Supplementary Figures

4.1. Supplementary Figure 1: Complexity matching in dyadic embodied social interaction. (A)

The distribution of inter-event intervals (IEI) contains the bin-averaged probability *P (IEI)* for each participant. **(B)** The relation between Allan Factor and window size for each participant averaged (SE) across all subjects and all sessions. **(C)** Complexity matching for all original dyad sessions (3 sessions x 10 dyads) and surrogate dyad sessions: t (29) = 2.4551, $p = 0.0102$.

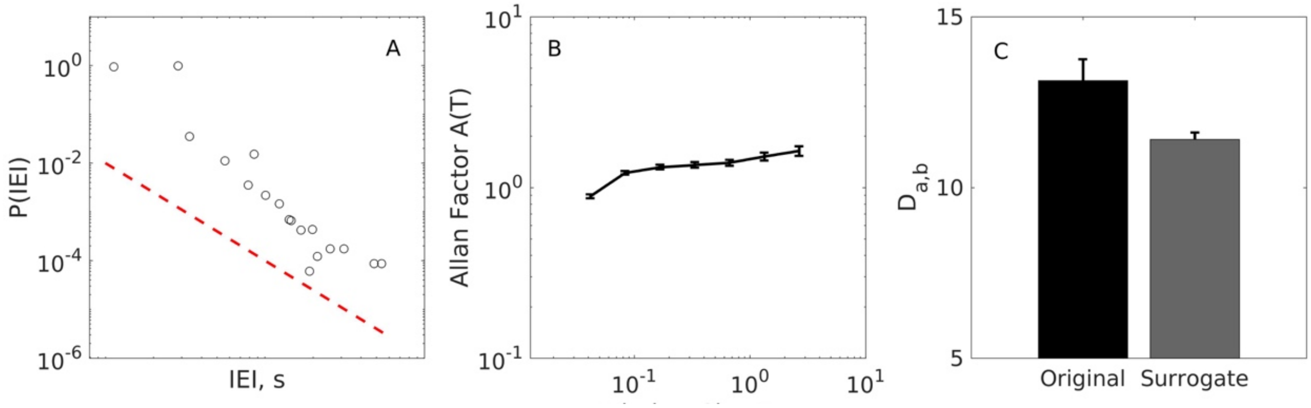

4.2. **Supplementary Figure 2: Positions and velocities for the members of one pair during one session.** Blue and red correspond to controls and high-functioning autism participants, respectively. The graphs show the pixels per time unit (20 milliseconds).

4.2.1. Position time series may be non-stationary, where the statistical moments change over time, in particular the mean making time-series analysis difficult given the presence of such trend.

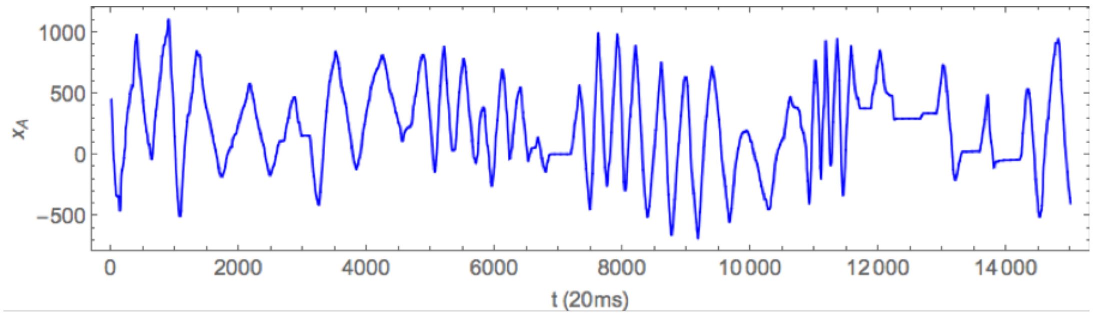

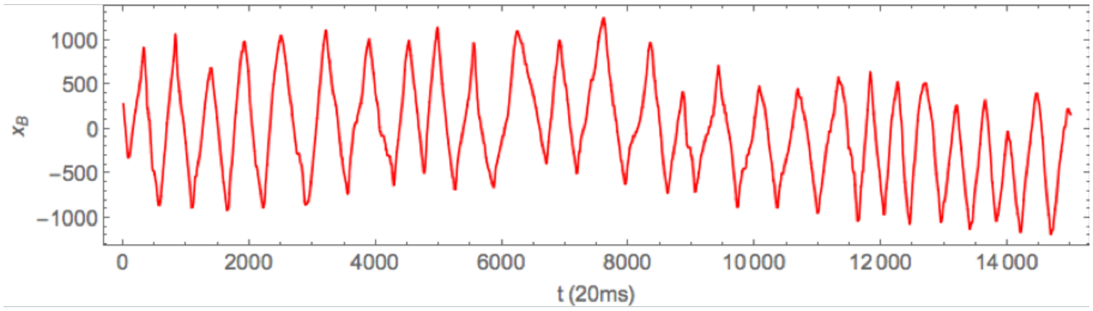

4.2.2. Velocity time series are stationary and oscillate around a fixed number (no trend). Velocity>0 moving right and velocity<0 moving left. Velocities are the differentiated values of the positions in the shown in the above graph.

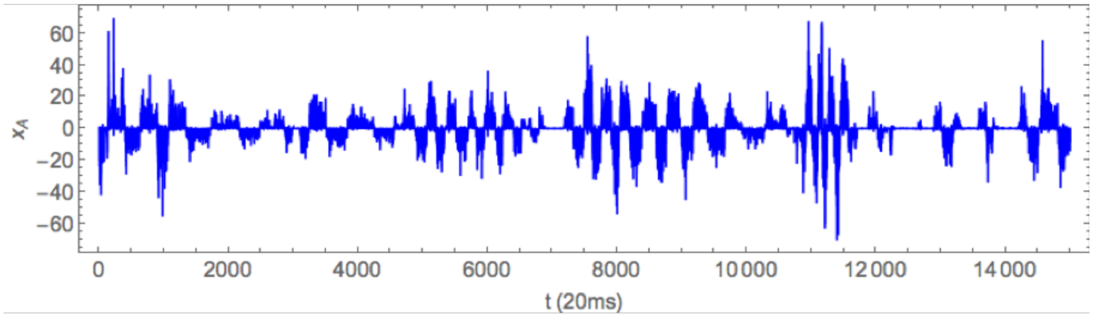

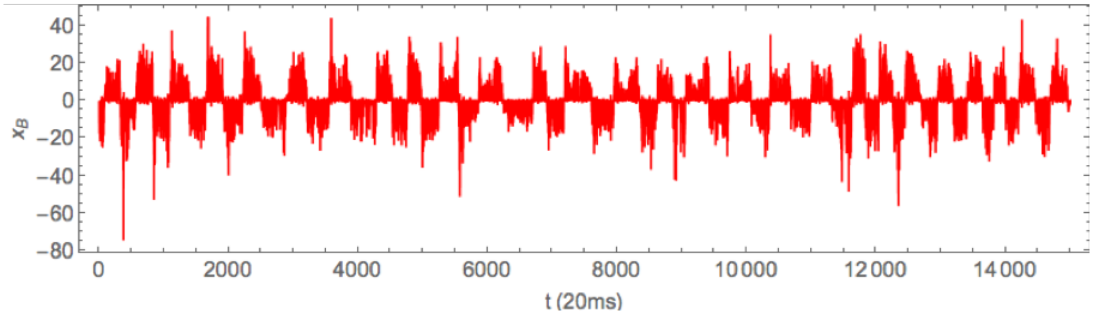

4.3. **Supplementary Figure 3: Example of coarse-grained analysis for a randomly taken velocity time series of one participant.** At coarser resolution scales, i.e., for larger r, variance tends to be less because small fluctuations are cancelled out and only the average behavior is left.

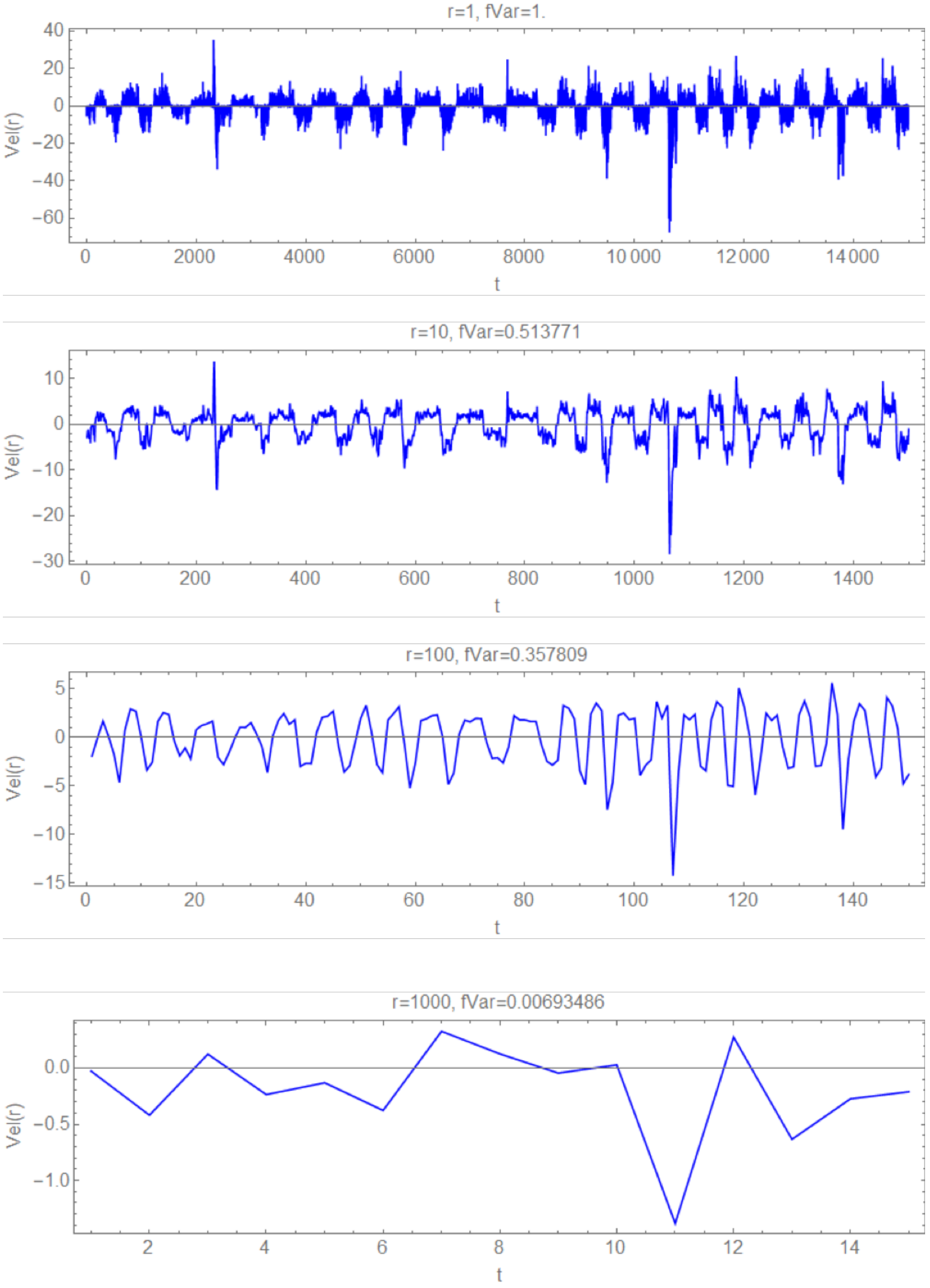

4.4. Supplementary Figure 4: Differences in fractional variance fσ^2 (*r*). Effect sizes and p-values for Mann-Whitney tests are plotted according to resolution factor *r*.

4.4.1. **Figure 4.1: HFA-CTRL differences per trial.** The type I error (α) threshold is set at 0.05. **A** and **C** correspond to Figure 2 of the main text's article.

A

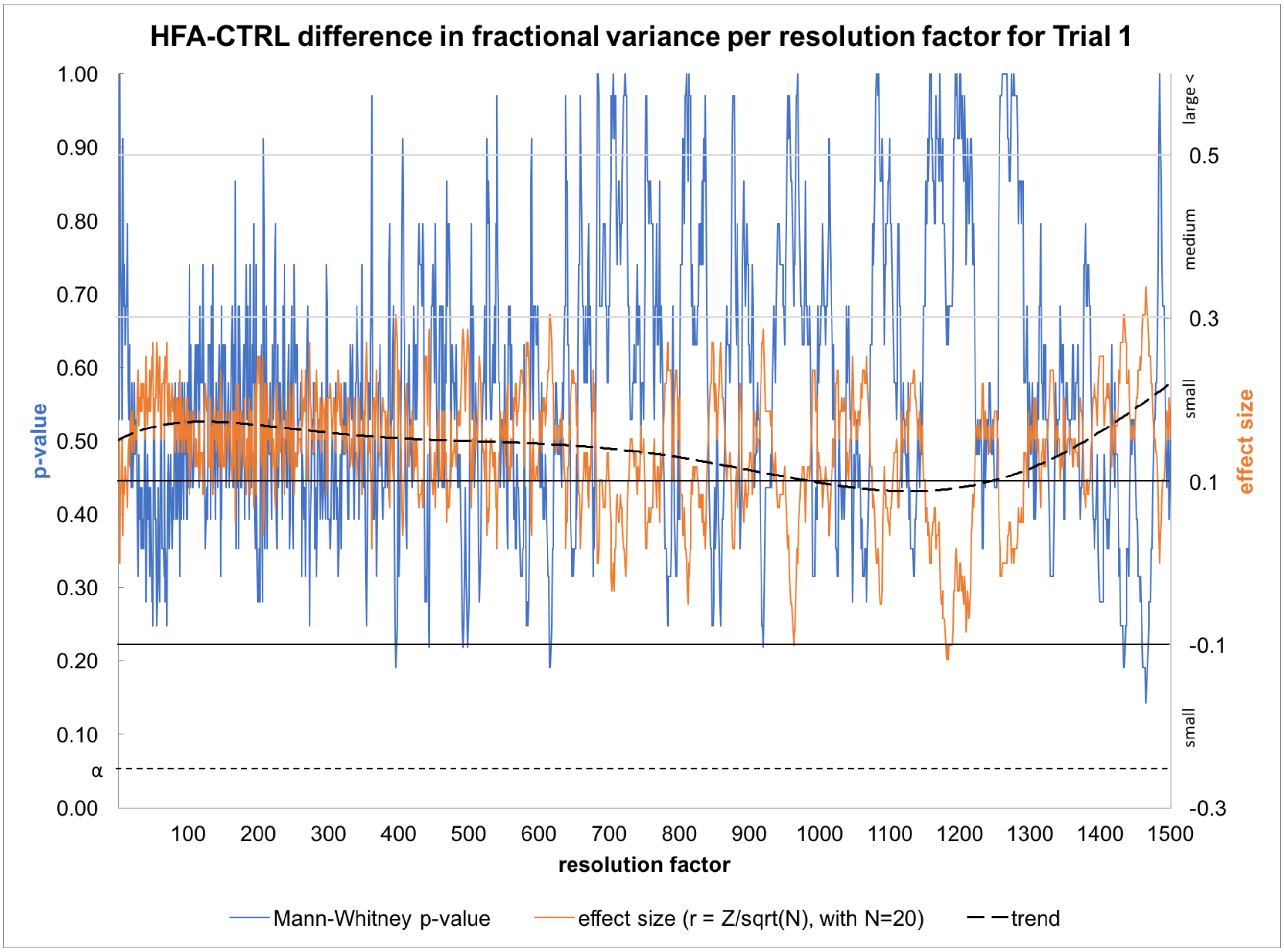

B

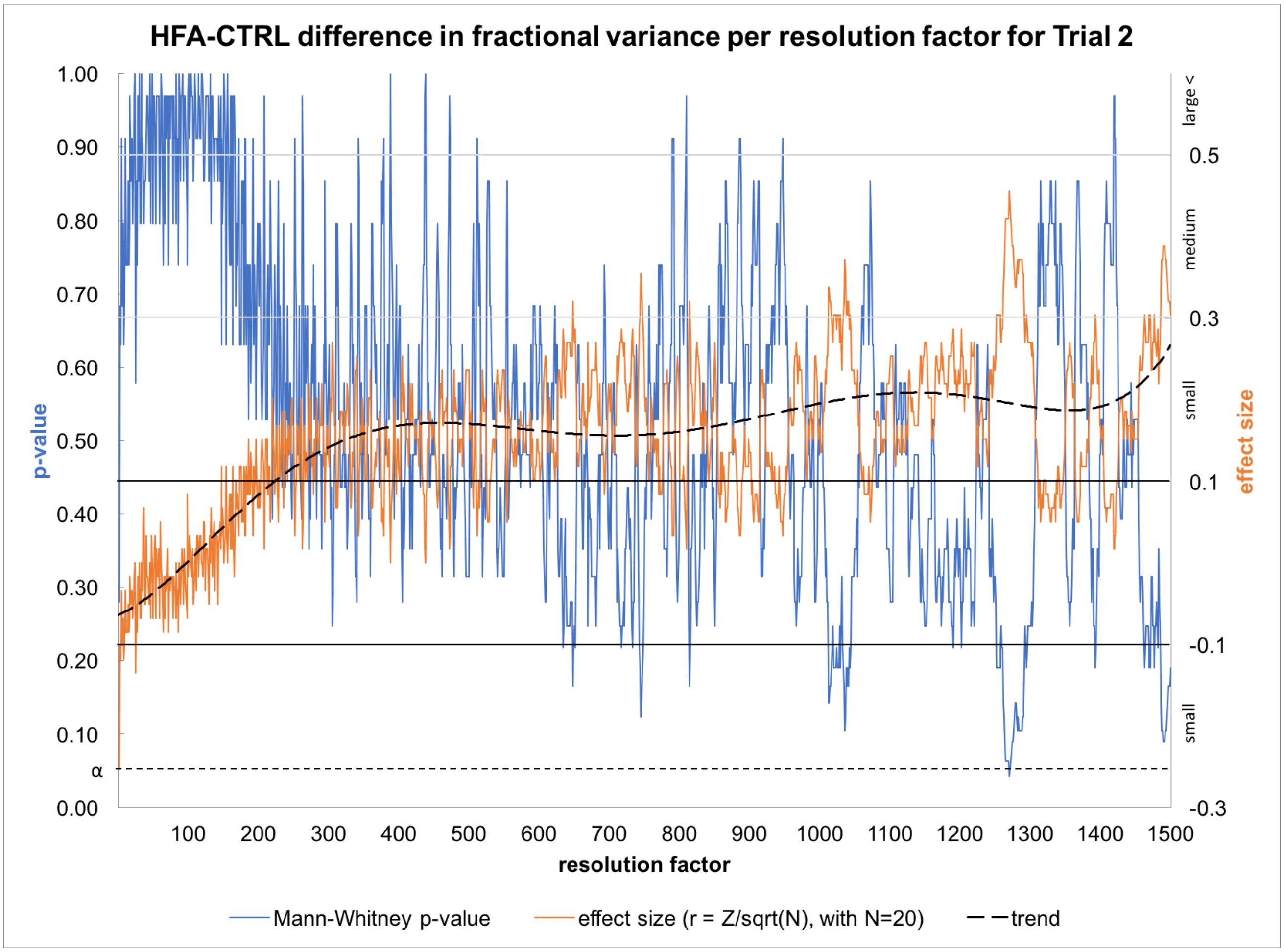
HFA-CTRL difference in fractional variance per resolution factor for Trial 2
p-value
1.00
0.90
0.80
0.70
0.60
0.50
0.40
0.30
0.20
0.10
α
0.00
100 200 300 400 500 600 700 800 900 1000 1100 1200 1300 1400 1500
resolution factor
effect size
0.5
0.3
0.1
-0.1
-0.3
large <
medium
small
small
Mann-Whitney p-value
effect size (r = Z/sqrt(N), with N=20)
trend

C

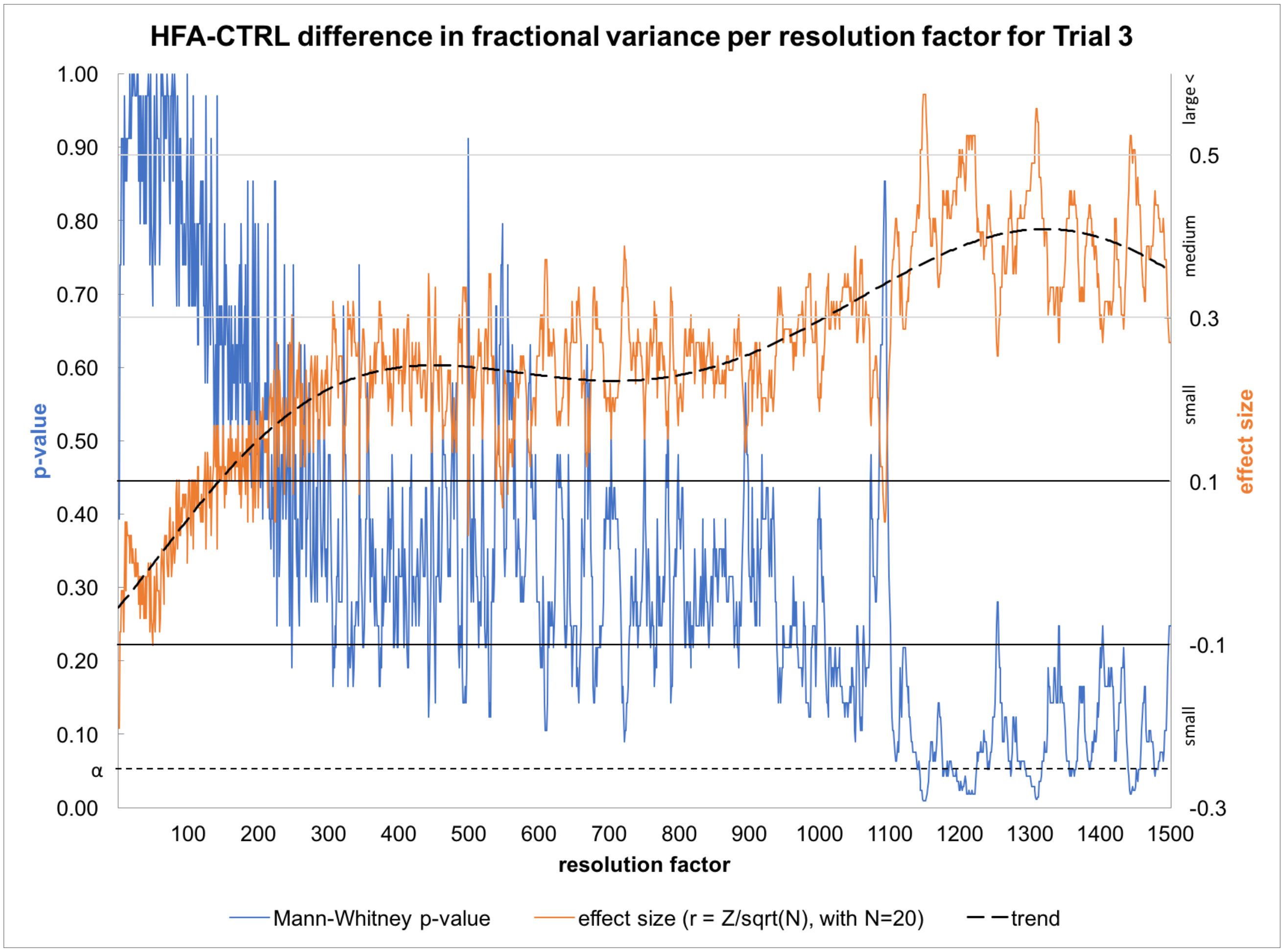

HFA-CTRL difference in fractional variance per resolution factor for Trial 3
p-value
effect size
1.00
0.90
0.80
0.70
0.60
0.50
0.40
0.30
0.20
0.10
α
0.00
large <
0.5
medium
0.3
small
0.1
-0.1
small
-0.3
100
200
300
400
500
600
700
800
900
1000
1100
1200
1300
1400
1500
resolution factor
Mann-Whitney p-value
effect size (r = Z/sqrt(N), with N=20)
trend

4.4.2. Figure 4.2: HFA vs CTRL changes from one trial to the next.

A

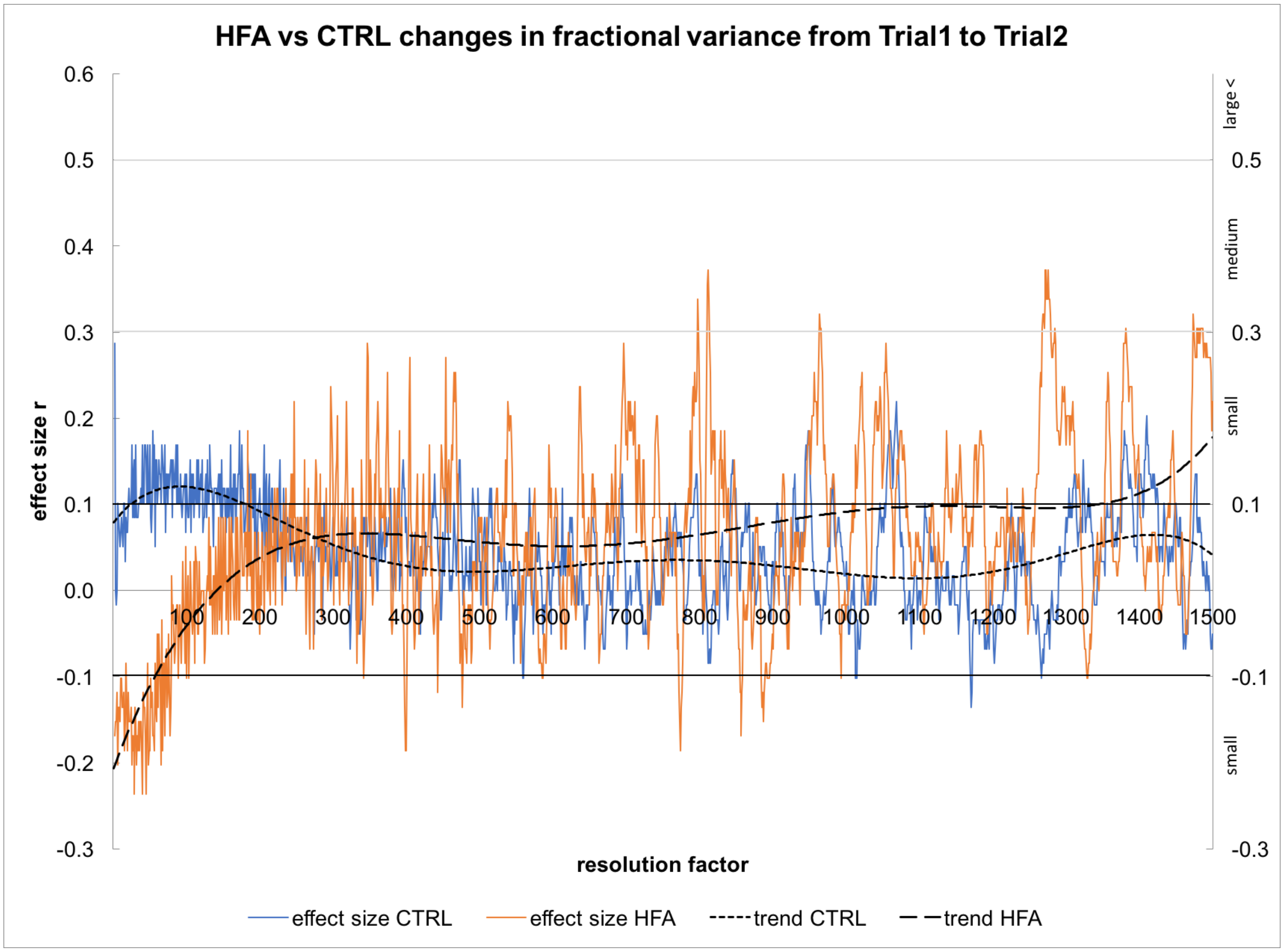

B

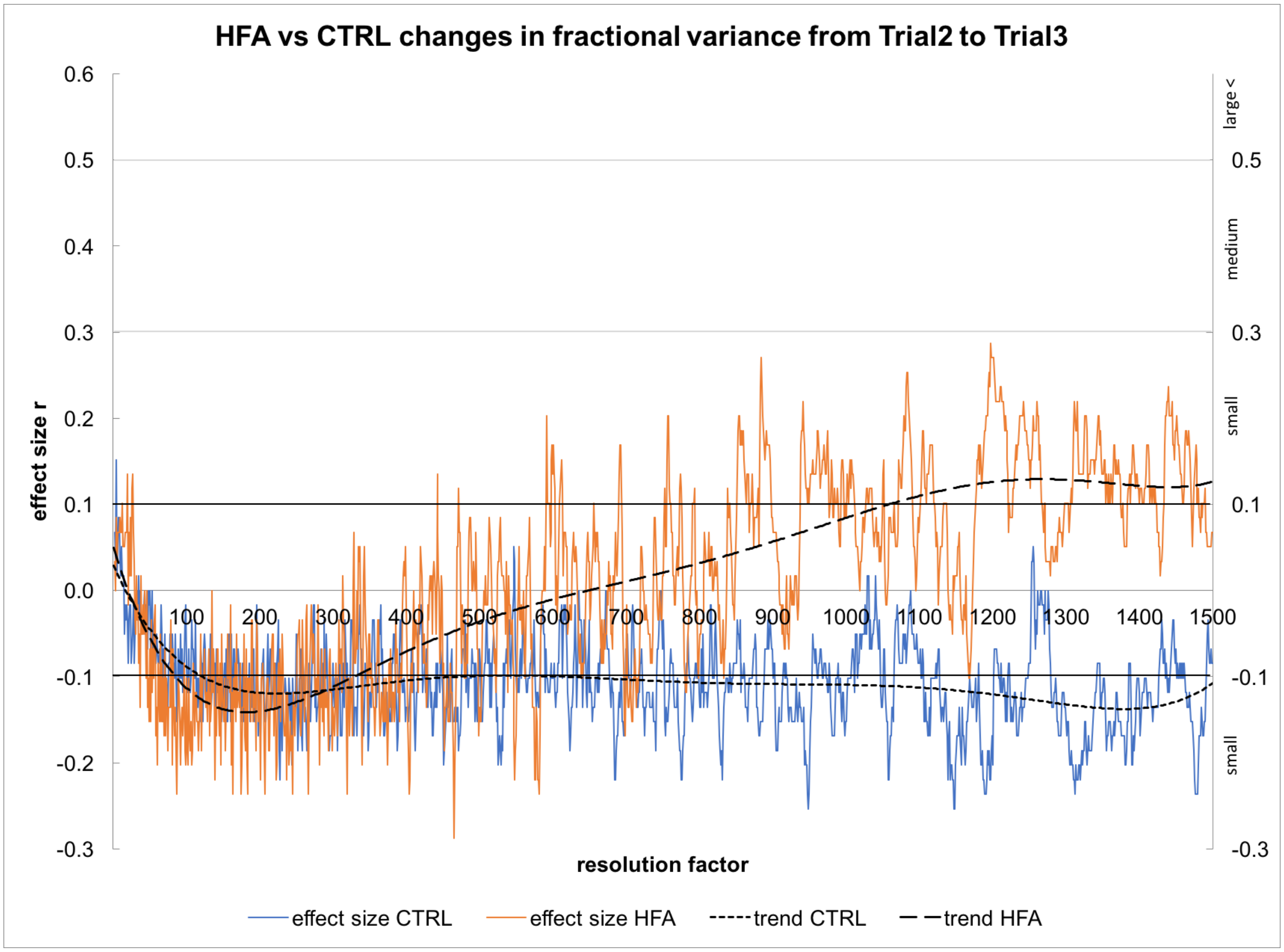
HFA vs CTRL changes in fractional variance from Trial2 to Trial3
0.6
0.5
0.4
0.3
0.2
0.1
0.0
-0.1
-0.2
-0.3
effect size r
100
200
300
400
500
600
700
800
900
1000
1100
1200
1300
1400
1500
resolution factor
large <
0.5
medium
0.3
small
0.1
-0.1
small
-0.3
effect size CTRL
effect size HFA
trend CTRL
trend HFA

C

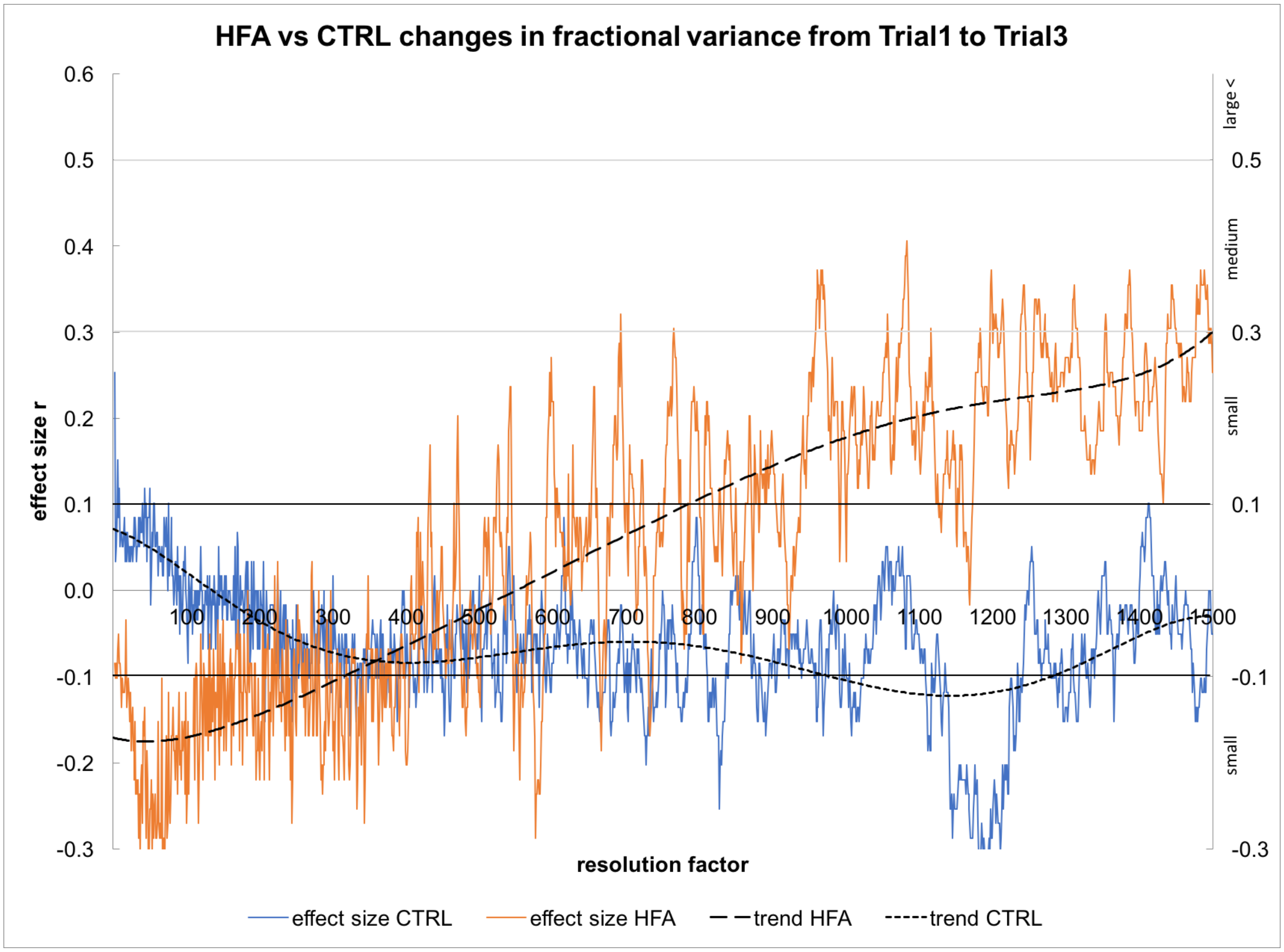
HFA vs CTRL changes in fractional variance from Trial1 to Trial3
effect size r
resolution factor
0.6
0.5
0.4
0.3
0.2
0.1
0.0
-0.1
-0.2
-0.3
100
200
300
400
500
600
700
800
900
1000
1100
1200
1300
1400
1500
large <
medium
small
small
effect size CTRL
effect size HFA
trend HFA
trend CTRL

5. Supplementary Table 1. Linear Mixed-Effects Models for Speed_Mean and Speed_SD.

The models are expressed by the equation $Y = \beta_0 + \beta_1 Trial + (\beta_1 + \beta_2) Trial*Group$. Bold font indicates significance.

Predictor	Speed_Mean			Speed_SD		
	β	*SE*	*t*	*β*	*SE*	*t*
Intercept	**2.1415*** **	**0.3624**	**5.909**	**3.9464*** **	**0.6678**	**5.910**
Trial	**0.6413***	**0.3033**	**2.114**	0.9188	0.5053	1.818
*Trial*GroupHFA*	-0.3024	0.1681	-1.799	**-0.5144***	**0.2268**	**-2.268**
Log likelihood†	-90.23			-118.03		
#Observations	60			60		
# Groups (Pairs)	10			10		
Variance (Pair)	0.23			0.13		
Variance (Residual)	0.25			0.54		

****** $p < 0.001$, ** $p < 0.01$, * $p < 0.05$***

†The goodness-of-fit test is with respect to the model standing previous in the series of models for the given outcome variables

6. Bibliography

- Fossion, R., Rivera, A. L., Toledo-Roy, J. C., Ellis, J., and Angelova, M. (2017). Multiscale adaptive analysis of circadian rhythms and intradaily variability: Application to actigraphy time series in acute insomnia subjects. *PLoS One* 12, e0181762. doi:10.1371/journal.pone.0181762.
- Singer, J. D., and Willett, J. B. (2003). *Applied longitudinal data analysis*. Oxford University Press doi:10.1093/acprof:oso/9780195152968.001.0001.
- Zapata-Fonseca, L., Dotov, D., Fossion, R., and Froese, T. (2016). Time-Series Analysis of Embodied Interaction: Movement Variability and Complexity Matching As Dyadic Properties. *Front. Psychol.* 7, 1940. doi:10.3389/fpsyg.2016.01940.
